# Towards a Rational Design of an Asymptomatic Clinical Herpes Vaccine: The Old, the New, and the Unknown

**DOI:** 10.1155/2012/187585

**Published:** 2012-03-26

**Authors:** Aziz Alami Chentoufi, Elizabeth Kritzer, David M. Yu, Anthony B. Nesburn, Lbachir BenMohamed

**Affiliations:** ^1^Laboratory of Cellular and Molecular Immunology, School of Medicine, University of California, Irvine, Irvine, CA 92697-4375, USA; ^2^Department of Immunology, Pathology and Clinical Laboratory Medicine, King Fahad Medical City, Riyadh 11525, Saudi Arabia; ^3^Institute for Immunology, School of Medicine, University of California, Irvine, Irvine, CA 92697-4120, USA; ^4^Chao Family Comprehensive Cancer Center, University of California, Irvine Medical Center, Irvine, CA 92868-3201, USA

## Abstract

The best hope of controlling the herpes simplex virus type 1 and type 2 (HSV-1 and HSV-2) pandemic is the development of an effective vaccine. However, in spite of several clinical trials, starting as early as 1920s, no vaccine has been proven sufficiently safe and efficient to warrant commercial development. In recent years, great strides in cellular and molecular immunology have stimulated creative efforts in controlling herpes infection and disease. However, before moving towards new vaccine strategy, it is necessary to answer two fundamental questions: (i) why past herpes vaccines have failed? (ii) Why the majority of HSV seropositive individuals (i.e., asymptomatic individuals) are naturally “protected” exhibiting few or no recurrent clinical disease, while other HSV seropositive individuals (i.e., symptomatic individuals) have frequent ocular, orofacial, and/or genital herpes clinical episodes? We recently discovered several discrete sets of HSV-1 symptomatic and asymptomatic epitopes recognized by CD4^+^ and CD8^+^ T cells from seropositive symptomatic versus asymptomatic individuals. These asymptomatic epitopes will provide a solid foundation for the development of novel herpes epitope-based vaccine strategy. Here we provide a brief overview of past clinical vaccine trials, outline current progress towards developing a new generation “asymptomatic” clinical herpes vaccines, and discuss future mucosal “asymptomatic” prime-boost vaccines that could optimize local protective immunity.

## 1. Introduction

Human herpes simplex virus type 1 and type 2 (HSV-1 and HSV-2) infections cause lifelong infections, with a spectrum of clinical manifestations including cold sores, genital ulceration, corneal blindness, and encephalitis [1–10]. Despite multiple approaches of therapy and prevention, HSV-1 and HSV-2 remain among the most common infectious viral pathogens of man. Current drug therapies such as oral acyclovir, valacyclovir, or famciclovir can treat herpes disease but do not prevent future attacks. Historically, many candidate vaccines that are effective on animal models of herpes infection turned unsuccessful in clinical trials [1, 11]. Sampling of past and ongoing vaccine trials is provided in Table 1. Progress towards an effective vaccine has stalled in the face of many unknown questions related to HSV-1 and HSV-2 infection and immunity. Namely, (i) the cellular and molecular mechanisms behind the failure of past herpes vaccines remain unknown; (ii) the cellular and molecular mechanisms that lead the majority of HSV seropositive individuals to be naturally protected exhibiting few or no recurrent clinical disease (designated as asymptomatic individuals), while other HSV seropositive individuals to have frequent ocular, orofacial, and/or genital herpes clinical episodes (designated as symptomatic individuals) remain unknown. Although the majority of individuals have few or no herpetic disease symptoms, they still continue shedding HSV all times. An efficient vaccine would not only relive the patients from herpetic diseases but also prevent virus reactivation and asymptomatic shedding. In the past, many vaccine immunotherapies have tried to stimulate the immune system against herpes, including about a dozen vaccines that reached mid- and late-stage clinical trials [1]. Every single one of these therapies had generated much excitement, but, for the most part, none of those therapies really did protect from herpes. Before devising more powerful treatments it is imperative to identify (i) the mechanisms underlying the suboptimal nonprotective immunity associated with natural infection, (ii) the major effectors of immunity that control each of the three phases of herpes infection (i.e., acute and latent), (iii) the sophisticated immune evasion strategies employed by HSV-1 and HSV-2 to dampen the immune response, (iv) the protective versus pathogenic protein (such as glycoprotein gK) Ag(s) among more than 80 immunogenic HSV proteins, and (v) a safe Ag delivery system.

Multiple review articles have adequately described and discussed the above issues [11–15]. The present paper focuses on bringing together past and recent published work that illuminates the current status of clinical herpes vaccine development. It presents an overview of our own vaccine approach to produce an “asymptomatic” herpes vaccine. *First, we describe the *common presentations of herpes simplex infections and diseases. *Second, *we portray the history of the different vaccine formulations that have led to the rationale for a herpes subunit vaccine. *Third*, we describe the process by which herpes protein Ags and derived “asymptomatic” epitopes suitable for inclusion in a multiepitope vaccine are being selected. *Fourth*, we shed new light on how an “asymptomatic” multiepitope lipopeptide vaccine can be designed to ensure optimal mucosal immunogenicity and discuss how, after prototype lipopeptide vaccines are designed, the program will move to the stage of clinical trials.

## 2. The Immunoepidemiology of Herpes

**Figure 1:**
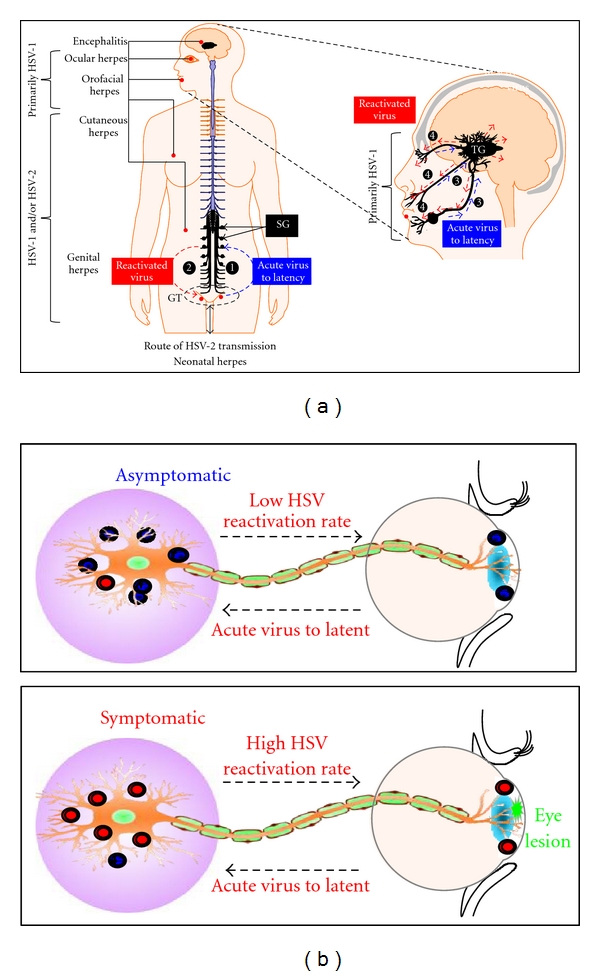
The majority of ocular herpes vaccines are injected parenterally, and although they induced strong systemic immune responses, they failed to generate significant local immune responses either in the eye or in trigeminal ganglia (TG). Local immune responses at these sites are likely needed to prevent virus transmission and to reduce virus replication, which should eventually reduce viral latency/reactivation and limit the severity of ocular herpes. Several results from our lab strongly suggest that there is linear association between presence of “asymptomatic” CD8^+^ T cells (bleu circles) in the TG and ocular mucosal immune system with the lack of eye disease. In contrast, the absence of asymptomatic CD8^+^ T cells and presence of symptomatic CD8^+^ T cells (red circles) may increase the rate of HSV reactivation and pathology. The upper panel shows scenario of an asymptomatic HSV-1 infection and the lower panel shows symptomatic HSV-1 infection and eye disease.

HSV-1 and HSV-2 are two closely related members of the Herpesviridae family and currently rank among the most prevalent infectious agents of man [16]. Several common presentations of herpes simplex infections and diseases are recognized. *Genital herpes:* although, both HSV-1 and HSV-2 account for herpes genitalis, a common sexually transmitted disease (STD), HSV-2 is more severe and has become more commonly associated with genital herpes [17, 18]. Currently, over 1 billion people around the world—one-sixth of the world population—are infected with genital herpes. In the USA alone, at least 40 to 60 million individuals have been infected by HSV-2. A therapeutic vaccine would ideally cure many of the adults who often suffer frequent recurrent outbreaks of genital herpes. In addition to the pain related to herpes ulceration, genital herpes causes a substantial psychosocial morbidity [18, 19]. Herpes genitalis contributes to a 2- to 4-fold increased risk of acquiring human immunodeficiency virus (HIV) [3, 20, 21] Genital herpes in HIV-infected individuals usually needs a longer duration of antiviral therapy along with continuation of highly active antiretroviral therapy (HAART). *Neonatal herpes:* in some cases, herpes infections are fatal to newborns and cause neonatal encephalitis [17, 22]. Genital herpes in late pregnancy increases the risk of neonatal herpes. Annually, a minimum of 2500 cases of neonatal herpes [23] and 3000 cases of herpes encephalitis result in significant morbidity and mortality in spite of antiviral therapies. *Ocular herpes: *ocular infection with HSV-1 is the leading cause of corneal blindness worldwide. The corneal scarring induced by herpes infection often leads to blindness, making HSV-1 a major cause of virus-induced blindness [4–6, 24–26]. Ocular infection with HSV-1 can cause other eye diseases ranging from blepharitis, conjunctivitis, and dendritic keratitis to disciform stromal edema and necrotizing stromal keratitis [27–30]. In the USA alone, over 400 000 people have a history of recurrent ocular HSV episodes requiring doctor visits, medication and, in severe cases, corneal transplants [28, 29]. Nearly 50,000 new and recurring cases are diagnosed each year. Shedding of reactivated HSV is estimated to occur at rates of 3 to 28% in adults who harbor latent HSV-1 in their sensory neurons [9–12]. However, the vast majority of these individuals do not experience recurrent herpetic disease and are designated “asymptomatic patients” [11, 14, 16]. In contrast, in some individuals (symptomatic patients), reactivation of latent virus leads to induction of ineffective or “symptomatic” HSV-specific CD4^+^ and CD8^+^ T cells [11, 16, 31] (Figure 1). Recurrent disease ranges from rare episodes occurring once every 5–10 years to outbreaks occurring monthly or even more frequently among a small proportion of subjects [16]. It is not known why ocular HSV-1 infection is asymptomatic in some individuals and symptomatic in others or why the frequency and severity of recurrences vary among symptomatic patients. The shedding rates in tears of asymptomatic individuals have been reported to be as high as 33.5% [13–16]. The immune mechanism(s) by which asymptomatic patients control herpes disease and symptomatic patients do not remains to be fully elucidated. *Orofacial herpes*: HSV-1-mediated recurrent facial herpes varies in severity. Symptomatic lesions usually occur on lips (cold sores), cheeks, within the nose, or on the nasal septum, which are painful and unpleasant [2, 32–34]. Dermal HSV infection can occur on any part of the body. Some oro-facial herpes are asymptomatic or appear as maculopapular lesions that may subsequently regress or develop into vesicular lesions, which then scab prior to healing. During asymptomatic or symptomatic outbreaks, HSV-1 is shed and can be transmitted to susceptible individuals.

The social and economic burden created by all types of herpes infection has set direct costs for treatment of these infections in the USA to over $400 million every year [16, 35]. Current drug therapies can treat the disease but do not prevent future viral attacks. Thus, novel strategies to treat, suppress, and prevent HSV infection are needed. An effective vaccine strategy remains the best hope for controlling the herpes pandemic. However, in spite of several clinical trials no vaccine has been proven sufficiently safe and efficient to warrant commercial development. It is imperative to know why past herpes vaccines have failed before we move towards developing a new vaccine strategy.

## 3. Past and Current Herpes Vaccines

Many classes of herpes vaccines and delivery systems have been attempted during the last century (Table 1). The following paragraphs review and discuss the approaches, the rationale, and the end results associated with some of the most widely studied herpes vaccines.

### 3.1. Inactivated and Replication-Defective Vaccines

 In the early 1920s the first vaccine was introduced as an inactivated virus, which was prepared from formalin-treated tissues of HSV-infected animals [36–38]. A heat- or ultraviolet-inactivated virus, grown in embryonic eggs, was proposed later (reviewed in [37, 39]). Subsequent vaccines strategies changed from inactivated to replication-defective HSV strains such as virulent type HSV mutants (i.e., lacking ICP8, ICP10, dl5-29, or VHS), discontinuously replicating virus known as “disabled infectious single cycle” or “DISC”, and a virus with a deletion of UL22, the late gene encoding glycoprotein H (gH) [40]. A DISC HSV-2 vaccine has entered clinical trials and has been found relatively safe with no serious adverse effects [41, 42]. Among HSV-seronegative subjects, dose-dependent induction of T-cell proliferation was noted four weeks after a single DISC HSV-2 immunization and continued for sixteen weeks after the second immunization. However, no responses were boosted in HSV-seropositive subjects. IFN-gamma (Th1) and IL-2 (Th2) production was also induced in HSV-seronegative persons in a dose-dependent fashion but not in HSV-seropositive persons. There was a lack of boosted IgG responses in both seronegative and seropositive subjects, indicating that DISC HSV-2 immunization might be shifted to Th1 responses. However, a recent immunotherapeutic phase II trial showed no clinical response in HSV-2 DISC-immunized persons [43].

### 3.2. Replication Competent Live Virus Vaccines

The replication competent live virus vaccine approach has the advantages of stimulating a broad immune response (antibody, CD4^+^ T cells, and CD8^+^ T cells) and presenting all epitopes from the entire genome to generate multiimmune responses. A case of recurrent human genital HSV-2 disease caused by a thymidine kinase-deficient, mouse-virulent strain has been reported [44]. Mutations in thymidine kinase gene do not attenuate HSV-2 replication sufficiently when used as vaccines. A second live attenuated HSV strain developed for vaccine use is RAV 9395 [45]. This virus was derived from HSV-2 strain G and contains deletions of both copies of the virulence factor g134.5, UL55, and UL56. Clinical results have not been reported for this mutant. The most extensive human studies are available with attenuated live HSV vaccine strain R7020, created by Branco and Fraser [46]. This virus was originated from HSV-1 strain F and is attenuated by a deletion extending from UL54 (encoding ICP27) through the promoter region of ICP4. In a dose escalation study, local reactions and systemic side effects were noted in HSV-1-infected persons.

### 3.3. Recombinant Viral Vectors

A number of trials have been pursued using recombinant live attenuated adenovirus and vaccinia recombinant viruses expressing HSV glycoproteins. These vaccines elicit Ag-specific CD8^+^ T cells after a single immunization [47–49]. Not surprisingly, no major vaccine company, in the USA or EU, is developing inactivated live vaccine candidates. An important lesson learned from the human live vaccine trials is the true feasibility (i.e., practicability) of a herpes vaccine.

The recent emergence of new concepts and technologies in biochemistry, genetics, and immunology has opened up the way to novel approaches in vaccine development. In the following paragraphs we bring together both the challenges and some recent progress made in developing a subunit herpes vaccine.

### 3.4. Plasmid (Naked) DNA Vaccines

The concept of using naked DNA [28] as a vaccine is to introduce herpes antigenic genes into dendritic cells (DCs) for endogenous processing and presentation to CD4^+^ and CD8^+^ T cells in draining lymph nodes or into other cells (e.g., epithelial cells) for cross-presentation by DCs, without the need for a viral vector. However, the competition within viral vector epitopes for endogenous processing reduced the efficacy. In addition the prior immunity to the viral vector and the potential dangers associated with a live virus are avoided when using DNA vaccines. Constitutive, tissue-specific promoters may be used for selective expression. The results of a number of plasmid DNA vaccine experiments in animal models of ocular and genital herpes have been reported [50]. The delivery of DNA vaccines usually requires high dosage of DNA plasmid to generate an immune response and often promotes Th_2_ response, which would not be expected to provide optimal protection against HSV infection and disease [50].

### 3.5. Recombinant Proteins-Based Subunit Vaccines

HSV has at least 11 enveloped glycoproteins that are expressed in infected cells. Among these, gB and gD glycoproteins are the most used immunogens since these are the dominant targets for neutralizing antibody production in HSV-infected individuals. gB and gD are attractive choices for subunit vaccines because they are the targets for both humoral (neutralizing and ADCC) and cell-mediated immunity (class I and class II restricted). gB and gD share high sequence similarity in HSV-1 and HSV-2 and may therefore provide cross-protection against both HSV-1 and HSV-2 infections.

Despite induction of high neutralizing serum antibody titers, the latest clinical vaccine trials, using recombinant protein gB and gD along with MF59 adjuvant, showed transient and partial protection [51–53]. More recently, intramuscular vaccination with a recombinant HSV-2 gD vaccine, using MPL as an adjuvant, protected ~70% of women who were HSV-1 and HSV-2 seronegative. However, there was no protection among men or among HSV-1 seropositive women [1, 53]. These results raised important questions regarding the role of gender-related factors and glycoprotein-based approach in vaccine efficacy. In this clinical trial, despite the induction of high neutralizing antibody titers that exceeded those of natural immunity, recurrent disease was not reduced suggesting that induction of a vigorous cellular immunity might be critical for therapeutic protection.

 Development of a herpes subunit vaccine has been motivated by previous successes achieved with other pathogens. However, major hurdles include identification of antigens that execute the specificity of immune system on HSV-1- and HSV-2-infected cells without harming uninfected cells. So far, early clinical trials indicate the need for the identification of target Ags, other than envelope glycoproteins gB and gD. However, this task is far from complete because of the large and complex herpes genome that encodes over 80 polypeptides, each of which could be a potential target to a protective immune effector. Tegument proteins are sandwiched in between the envelope and capsid proteins of HSV and have been reported to be major targets for T-cell responses. A recent human study that utilized pools of overlapping synthetic peptides presented to CD8^+^ T cells through autologous dendritic cells showed that the responses to individual open reading frames (ORFs) ranged from ≤5% to a maximum of 70%. Interestingly, the highest responses detected in seropositive individuals were focused on six tegument proteins: UL39, UL25, UL27, ICP0, UL46, and UL47 in descending order. These six tegument proteins are therefore considered to be the best candidates for T-cell-based vaccines [54]. Whether the T-cell responses of asymptomatic versus symptomatic individuals to these tegument proteins are similar or different remains to be determined.

Other current vaccine strategies, listed in Table 2, include the use of virus-like particles (VLPs), adenoviral vectors, and lipopeptide vaccines; however, very few have been approved for human use. Two promising approaches to herpes vaccination are currently being pursued in our and others laboratory based on entirely different theoretical approaches. The first approach is the subunit vaccines that use “asymptomatic” epitopes from envelop, tegument, and/or regulatory proteins with or without adjuvants [2, 32]. The second approach is the genetically engineered live attenuated vaccine without any putative neurovirulence or immuno-evasion genes [39, 55, 56]. Few vaccines with these dual modalities have attempted to provide sterilizing immunity. Nevertheless, prior attempts at HSV subunit and genetically engineered live attenuated vaccines have offered important lessons for the design of clinical and preclinical studies to evaluate vaccines of this kind.

## 4. T-Cell-Inducing Herpes Simplex Vaccines—What Is the Future

Shedding of reactivated HSV-1 and HSV-2 that leads to recurrent herpetic disease is estimated to occur at rates of 3 to 28% in adults who harbor latent virus in their sensory neurons [57–60]. Recurrent disease ranges from rare episodes occurring once every 5–10 years to outbreaks occurring monthly or even more frequently among a small proportion of “symptomatic patients” [61]. For simplicity, one can categorized seropositive individuals based on the frequency of their recurrent disease into two major groups: (1) the symptomatic individuals (with a history of recurrent corneal, genital, and/or orofacial herpetic disease) and (2) the asymptomatic individuals (never had any recurrent herpes disease, ocular, genital, orofacial, or otherwise). The vast majority of seropositive individuals do not experience recurrent herpetic disease and are designated “asymptomatic” [59, 61, 62]. In contrast, in “symptomatic” individuals reactivation of latent virus leads to mild to severe herpetic disease [59, 61, 63]. It is not known why HSV-1 and HSV-2 reactivation/shedding is asymptomatic in some individuals and symptomatic in others or why the frequency and severity of recurrent disease vary among symptomatic individuals. Interestingly, for genital herpes, symptomatic and asymptomatic patients shed the virus at similar rates [59, 64]. It is likely to be the same for ocular herpes, since shedding rates in tears of asymptomatic individuals has been reported to be as high as 33.5% [61, 62, 65, 66]. The immune mechanism(s) by which asymptomatic patients control herpetic disease and symptomatic patients do not remains to be fully elucidated [67]. Identifying these mechanisms, or at least the viral antigens (Ags) and epitopes involved, is critical to understanding how to protect against recurrent herpetic disease and for rational advances in therapeutic vaccine development. In the most recent clinical vaccine trials [1], despite recombinant-proteins-based HSV-2 vaccines induced neutralizing antibody titers that exceeded those produced by natural immunity, neither symptomatic infections nor symptomatic recurrences were affected by therapeutic vaccination. This suggests that induction of vigorous cellular immunity is critical for better protection [68, 69]. Thus, T cells appeared to be an important part of naturally acquired protective immune responses against herpetic disease, and inducing “asymptomatic” T cells by vaccination has dominated much of our research effort.

 It is likely that Ag exposure during long-term herpes simplex infections may shape different T-cell repertoires over time, in symptomatic and asymptomatic individuals. The unique epitope-specific T-cell repertoire of each symptomatic and asymptomatic individual, known as “private specificity” [70–72], is thought to regulate whether herpes reactivation will result in viral control, asymptomatic persistence, or severe disease. Thus, in symptomatic individuals, reactivation of latent virus leads to induction of ineffective or “symptomatic” HSV-specific CD4^+^ and CD8^+^ T cells [59, 61, 63]. In contrast, in asymptomatic individuals, reactivation of latent virus leads to induction of protective or “asymptomatic” HSV-specific CD4^+^ and CD8^+^ T cells [59, 61, 63]. A good starting point for the development of an efficient therapeutic herpes vaccine would be to identify the matrices of protective or “asymptomatic” Ags and epitopes strongly recognized by T cells from asymptomatic individuals. Our recent findings support the idea that symptomatic and asymptomatic individuals have different levels of HSV-specific T-cell repertoires ([67, 73–75], Dervillez, submitted). We found that T cells from symptomatic and asymptomatic individuals, with similar HLA, have dramatically different profiles of responses to HSV epitopes. A set of human T-cell epitopes from HSV-1 glycoproteins B and D (gB&gD) are strongly recognized by T cells from HSV-1-seropositive asymptomatic individuals, but not by T cells from symptomatic individuals [67, 73–75]. In contrast, a different, nonoverlapping set of gB and gD epitopes are strongly recognized by T cells from symptomatic but not by T cells from asymptomatic individuals. However, this difference is not due to clonal T-cell deletion since there is not a complete lack of T-cell response. The “asymptomatic” T-cell precursor appears to exist in symptomatic patients and vice versa.

 Our preclinical vaccine trial in “asymptomatic” HLA transgenic (HLA Tg) rabbits showed that immunization with asymptomatic human CD8^+^ T-cell epitopes from HSV-1 gD induced strong human epitope-specific CD8^+^ T cell responses and reduced HSV-1 shedding in tears and corneal disease following an ocular challenge [34]. Rabbits support spontaneous reactivation of HSV-1 at a level similar to humans (~10%). Similarly, the rate of recurrent corneal disease in rabbits is also similar to that of humans. Unfortunately this rate is very low (<1% of eyes). However, we have been able to vaccinate HLA transgenic rabbits that developed recurrent corneal disease (i.e., a “symptomatic” HLA transgenic rabbits). One rabbit did develop a modest T-cell response against the “asymptomatic” peptides following vaccination. This suggests that symptomatic individuals will be able to respond appropriately to a therapeutic asymptomatic epitope-based vaccine and develop asymptomatic CD8^+^ T-cell responses specific to the asymptomatic epitopes. Despite “seeing” both “asymptomatic” and “symptomatic” epitopes (through virus exposure), the vaccinated asymptomatic individuals may not appear to revert to mixed T-cell populations but rather develop mainly the protective asymptomatic responses. The results also provide tangible preclinical evidence that immunization of “symptomatic” individuals with an “asymptomatic” epitope-based vaccine will likely boost “asymptomatic” T-cell responses in symptomatic patients (as it did in HLA Tg rabbits) and that may be sufficient to stop or reduce recurrent disease, upon encounter with the virus, through reinfection or reactivation of latent virus. In contrast, a therapeutic vaccine containing whole virus or whole viral proteins would be expected to induce symptomatic as well as asymptomatic CD8 T-cell responses, thus boosting harmful as well as protective immunity. Obviously, boosting harmful immunity should be avoided. This can be accomplished using an asymptomatic epitope-based therapeutic vaccine.

 Since we have previously shown that there is significant HSV-1-specific CD8 T-cell exhaustion during latency in mice, it may be useful to complement the therapeutic asymptomatic epitope vaccine strategy with exhaustion-pathway blockage. This is likely to result in an even stronger CD8^+^ T cell response in latently infected “symptomatic” individuals.

## 5. The New Vaccines: Multivalent “Asymptomatic” Lipopeptide Vaccines

It has been demonstrated that immunizations with a single immunodominant CD8^+^ CTL epitope, administered in a suitably strong adjuvant, can protect MHC-haplotype-identical inbred mice against genital herpes [31, 76]. The MHC-haplotype-outbreed nature of the human population obviously complicates the development of single peptide-based vaccines. Bearing in mind the particular properties that would be required in a prospective human peptide vaccine, we conceived a strategy in which virus-specific CD4^+^ and CD8^+^ T cell responses could be generated in different haplotypes using a single or a mixture of lipopeptide vaccines [2, 34, 77, 78].

Though subunit vaccines with a combination of protective epitopes are promising, several challenges are still associated that need to be addressed. (i) Adding many epitopes together in a cocktail can lower the dose of each one, thereby reducing overall efficacy. (ii) Some balances during epitope selection must be considered in order to deal with the highly variable MHC-haplotype human population so that the immunogenicity and protection are not impaired or lost. (iii) Since both antibody and cell-mediated responses are necessary for full protection, it is important to control which epitopes stimulate which type of response. In spite of these challenges, we believe that among the current subunit vaccine types, a multiepitope peptide vaccine is best suited to provide the complex epitope combination necessary to protect a wide variety of human populations.

**Figure 2:**
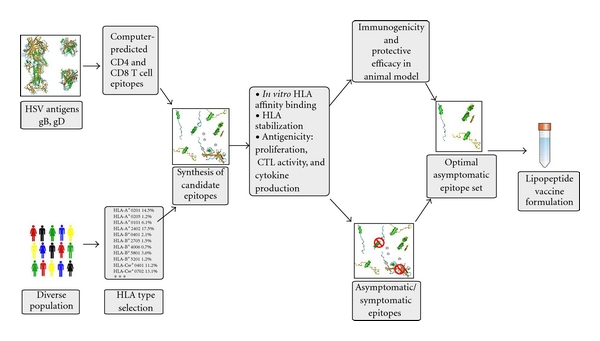
*Illustration of steps in developing an asymptomatic lipopeptides-base herpes vaccine. *The lipopeptide vaccine formulation is developed following multistep strategy. This starts from the identification of a symptomatic and asymptomatic herpes population and highly immunogenic HSV proteins. Next, asymptomatic CD4^+^ and CD8^+^ T-cell epitopes are discovered and covalently linked to a TLR2 agonist (Palmitic acid) leading to self-adjuvanting lipopeptides [12].

In other systems, induction of simultaneous responses against multiple epitopes derived from multiple Ags has already been demonstrated. The immunogenicity of multiepitope constructs appears to be strongly influenced by a number of different variables, and the immunogenicity (or antigenicity) of the same epitope expressed in the context of different vaccine constructs can vary over several orders of magnitude. This situation underscores the necessity of a systematic study of different variables in order to establish clear criteria for the optimal design of multiepitope vaccines (reviewed in [79, 80]). To address this in the context of herpes, we are designing and optimizing multiepitope vaccines comprising a panel of CD8^+^ and CD4^+^ T-cell epitopes derived from major herpes Ags as described in Figure 2. These epitopes were identified by class I and class II algorithm predictions and peptide binding/recognition strategies and recognized by recall immune responses from seropositive individuals as well as from HLA transgenic mouse models [24]. Studies need to optimize the vaccine efficacy by (i) eliminating junctional epitopes and spaces between epitopes, (ii) the effect of flanking regions, and (iii) cellular targeting to Ag processing and presentation pathways. Recognition of individual epitopes is demonstrated by immunogenicity assays utilizing HLA transgenic mice and/or antigenicity assays using human APCs transfected *in vitro* with the prototype vaccine. The simplest vaccine configuration capable of effective delivery of the selected sets of epitopes will also be determined. Subsequent studies will identify the optimal vaccine delivery strategy for simultaneous induction of immune responses against multiple epitopes and the appropriate vaccine formulation. Overall, it is anticipated that these studies will define operational rules for the design and optimization of multiepitope-based vaccines.

**Figure 3:**
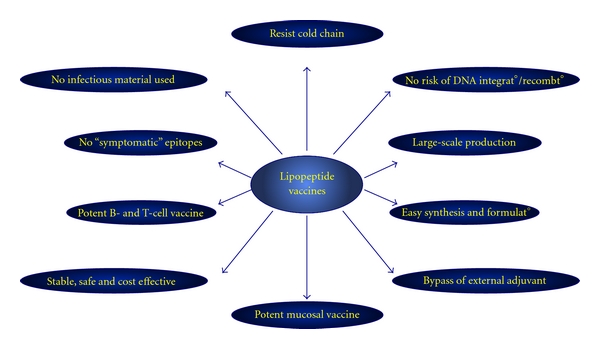
A representative diagram showing the advantages of lipopeptide-based vaccines strategy, as elaborated in the text (see Section 6).

The profile of HSV antigens presented during different phases of herpes infection implies that an ideal vaccine must be multivalent and capable of inducing multiimmune responses. Since the first demonstration of the technology, a few years ago, lipopeptide vaccines have emerged as a promising method of vaccination. In a variety of experimental systems, lipopeptide vaccines have been shown not only to induce potent immune responses but also to offer many advantages in terms of ease of construction, testing, and production (Figure 3). In the following paragraph we summarize the progress achieved in developing a lipopeptide-based vaccine that protects a progestin-induced susceptible mouse model of genital herpes from infection following intravaginal infection with either HSV-1 or HSV-2. We describe initial studies of immunogenicity and outline the strategies being employed to design the next generation of lipopeptide vaccines.

A presumed advantage of lipopeptide immunogens is the possibility of producing multivalent vaccines by a simple physical mixture and simultaneous delivery of lipopeptides bearing epitopes derived from one or more Ags, rather than chemical covalent association of T-cell epitopes in one molecule [81, 82]. Besides easy construction, such a mixture may also prove more effective than separate vaccines for each epitope. These results in mice coincide with a recent clinical trial of HIV-1 vaccine with similar strategy. The results showed that up to six T-cell lipopeptides, selected from three different HIV proteins (Gag, Nef, and Env) delivered simultaneously as a cocktail, were strongly immunogenic in humans [81, 83]. These findings are also in line with a recent report showing that immunization with a mixture of six lipopeptides derived from four malaria Ags is effective in inducing multispecific CD4^+^ Th_1_ cells, CD8^+^ CTLs, and IgG responses in nonselected “outbred” human populations [81, 82].

Shortcomings in developing an effective immunization strategy against genital herpes include an imperative requirement for a safe Ag delivery system [39]. In most cases, unmodified nonvectorized peptide Ags fail to elicit virus-specific T cells, unless they are attached to a carrier protein or delivered with a strong adjuvant [84–86]. Often, the delivery of peptides in this manner is unsafe and/or promotes Th_2_ responses [87, 88], which would not be expected to provide optimal protection against HSV infection [89]. Lipid tailing of peptides offers a safe formulation that generates CD4^+^ T cells, CD8^+^ T, cells and Ab responses in the absence of any adjuvant, apart from the lipid moiety itself [81, 83, 90–94].

Physicochemical safety and immunogenicity studies in animal models and in two human phase I clinical trials have established the safety and efficacy of HIV and malaria lipopeptide vaccine candidates [81, 83, 90–94]. The present paper focuses on herpes lipopeptides and demonstrates their safety and ability to induce CD4^+^ Th_1_ cell-dependent protective immunity against genital herpes when delivered in water via a parenteral route. Since HSV-1 and HSV-2 invade human mucosa, delivery of Ags through the IVAG route would induce better protection against these sexually transmitted infections [95, 96]. We previously demonstrated that intranasally administered lipopeptide epitopes induce both mucosal and systemic B- and CD4^+^ Th_1_-cell responses [2, 32–34, 97–100]. Similar results were obtained using the human cytomegalovirus pp65-derived CD8^+^ CTL lipopeptides in which higher levels of virus-specific CTL were obtained with lipopeptide delivered mucosally [2, 32–34, 97–101]. Assessing the immunogenicity and protective efficacy of HSV-1 and HSV-2 lipopeptides following administration through the IVAG or other mucosal routes (e.g., topical ocular, sublingual, intranasal, or intrarectal) is being pursued in our laboratory and will be addressed in future paper.

### 5.1. Advantages of Asymptomatic Epitope-Based Vaccines

1. exclusion of potentially harmful symptomatic epitopes,
2. focused immune response against immunodominant and protective asymptomatic epitopes,
3. molecularly defined-no immunoevasion or pathogenic molecules.

Our lipopeptide vaccine construct is molecularly defined, which makes it a particularly advantageous approach compared to other vaccine strategies (see Tables 1 and 2). Because the lipopeptide vaccine is constructed of chosen asymptomatic epitopes, we are able to exclude symptomatic epitopes that would otherwise reduce its efficacy or the harmful side effects. The “symptomatic” epitopes may direct T-cell responses away from those that are best suited to clear the viral infection with minimal pathogenic reaction (Figures 2 and 3 and [5]). An immunopathogenic T-cell response might occur through stimulating low-affinity oligoclonal responses that inhibit broad-based T-cell responses to other well-presented high-affinity epitopes, thus deviating protective responses to damaging responses. While protein-based vaccines contain both symptomatic and asymptomatic epitopes from the same protein, our lipopeptides exclusively contain CD4 and CD8 asymptomatic epitopes from one or many herpetic proteins [12]. Because symptomatic epitopes can have pathological effects when used in a vaccine, our group has made it a priority to identify the asymptomatic epitopes of HSV glycoproteins, tegument proteins, and regulatory proteins [33, 67, 74, 77]. The lipopeptide vaccine has the fewest side effects compared to the majority of vaccine strategies so far used in clinical trials [102] (Table 2). In addition, while all the other vaccines induce a variety of specific CD4^+^ and CD8^+^ T cell responses including low-affinity ones, lipopeptides induce a focused, strong and long-lasting CD4^+^ and CD8^+^ T cell response against the selected immunodominant asymptomatic epitopes only [12, 32]. The lipopeptide vaccine strategy also excludes those HSV proteins that may enable the virus to evade the host immune system. While live attenuated virus vaccine, inactivated virus vaccine, or protein-adjuvant based vaccines contain unknown and potentially harmful molecules, lipopeptide vaccines are molecularly defined and do not contain pathologic molecules, such as ICP-47 [103–105].

### 5.2. Mucosal Route of Vaccination

Mucosal surfaces constitute an impressive first-line defense that is frequently exposed to HSV-1 and HSV-2 infections [106–109]. The mucosal immune system is largely separate and distinct from the systemic immune system [106–108] and is more complex [106–108]. The tissue compartments involved in mucosal immunity are mucosal inductive sites and mucosal effector sites. The inductive sites are comprised of lymphoid tissue, where the triggering of naïve immune cells and the generation of memory-effector cells take place. This is where Ags are encountered, taken up by APCs, processed, and presented to B and T cells, which may then migrate to effector sites where immune T cells function [106–108]. Mucosal tissues mostly contain DCs, which have properties to optimize Ag uptake, processing and T-cell stimulation [110–115]. Mucosal subunit vaccines are designed for needle-free application, therefore safe and cost effective compared to other vaccines. Efficacy of mucosal vaccine has been well established for the oral poliovirus vaccine, but today very few other vaccines administered by the mucosal route are available commercially. Tremendous research efforts have significantly improved the classical approach used to create these vaccines, and alternative methods of immunization based on new concepts of mucosal immunity are being developed.

## 6. The Unknown

 Progress towards an effective vaccine has stalled in the face of many unknown questions and related to HSV-1 and HSV-2 infection and immunity. Namely, (i) the cellular and molecular mechanisms behind the failure of past herpes vaccines remain unknown, (ii) the cellular and molecular mechanisms that lead the majority of HSV seropositive individuals (i.e., asymptomatic individuals) to be naturally “protected” exhibiting few or no recurrent clinical disease while other HSV seropositive individuals (i.e., symptomatic individuals) to have frequent ocular, orofacial, and/or genital herpes clinical episodes remain unknown, (iii) HSV-specific CD8^+^ T cells, selectively activated and retained in latently infected trigeminal and sacral ganglia [34, 74, 116, 117], play a crucial role in suppressing full blown reactivation of HSV-1/2 latency [103, 116], apparently by interfering with virus replication and spread following the initial molecular events of reactivation. Thus, rather than completely eliminating the latent HSV-1 from trigeminal and sacral ganglia, reactivations appear to be “kept in check” by CD8^+^ T cells [12, 12, 74, 118]. The importance of CD8^+^ T cells in providing constant immunosurveillance of latently infected neurons, in which the virus starts to reactivate, is suggested by numerous mouse, guinea pig, rabbit, and human studies [34, 116, 119–122]. However, it is still unclear why and how the virus manages to sporadically escape CD8^+^ T cell-mediated immunosurveillance and efficiently reactivate from latency to often cause ocular, orofacial, and genital herpes diseases. Identification of the immune evasion mechanism used with HSV-1 and HSV-2 would certainly help develop stronger preemptive immunotherapeutic vaccine strategies against herpes.

 In the past, dozens of vaccine immunotherapies have tried to stimulate the immune system against herpes, including about a dozen vaccines that reached mid- and late-stage clinical trials. Every single one of these therapies has generated much excitement, but, for the most part, none of those therapies really did protect against herpes. Before devising more powerful treatments it is imperative to identify (i) the mechanisms underlying the suboptimal nonprotective immunity associated with natural infection, (ii) the major effectors of immunity that control each of the three phases of herpes infection (i.e., acute and latent), (iii) the sophisticated immune evasion strategies employed by HSV-1 and HSV-2 to dampen the immune response, (iv) the protective versus pathogenic protein Ag(s) among more than 80 immunogenic HSV proteins, and (v) a safe Ag delivery system.

 Our laboratory is hoping to bridge some of the gaps in our knowledge including (i) why CD4^+^ and CD8^+^ T cells from asymptomatic and symptomatic individuals tend to recognize different sets of nonoverlapping HSV Ag epitopes; (ii) Are the epitopes recognized by CD4^+^ and CD8^+^ T cells from asymptomatic individuals protective against virus replication, herpetic disease, and/or latent infection? (iii) Can the magnitude of CD4^+^ and CD8^+^ T-cell responses to “asymptomatic” human T-cell epitopes be significantly improved by epitope enhancement (increasing HLA binding affinity) or increasing their bioavailability (increase resistance to proteolysis)? (iv) Can the combination of “improved asymptomatic T-cell epitopes” broaden the ocular immune responses? (v) Can a multivalent lipopeptide vaccine, bearing combination of “improved symptomatic CD4^+^ and CD8^+^ T-cell epitopes,” delivered intranasally, topically to the eyes, or intranasally induce robust local immunity? (vi) Can local HSV-specific immunity induced at the sites of infection (i.e., the eye, the genital tract, trigeminal ganglia, and sacral ganglia) or in the draining lymph nodes prevent virus transmission/reactivation and/or limit the severity of ocular and genital herpes?

A targeted immunotherapeutic vaccine is necessary to induce robust localized immune responses (i.e., in central nervous system, spinal cord, trigeminal ganglia, and sacral ganglia), to quell virus replication, drive the pathogen into a “latent” state, and likely hinder viral reactivation. However, an immune response in the central nervous system might not be good. The release of inflammatory mediators including reactive oxygen species may cause cell death in the central nervous system (CNS). However the death from HSV-1-mediated frank sporadic encephalitis is a rare event. A good understanding of the contribution of resident and infiltrating leukocytes within the nervous system in response to herpes infection is necessary to identify candidate “asymptomatic” epitopes and immune molecules, which do not induce unwarranted inflammation coinciding with the maintenance of the antiviral state.

We believe that in the next five years research should focus on (1) identifying more “asymptomatic” versus “symptomatic” herpes epitopes, (2) qualitatively and quantitatively analyzing T cells in symptomatic versus asymptomatic patients that could break new ground in our understanding of the immune mechanisms underlying herpes pathogenesis in humans, (3) incorporating only promiscuous “asymptomatic” epitopes into vaccines, (4) using mucosal vaccine strategies, such as lipopeptides, to immunize against herpes, and (5) Using “humanized” susceptible HLA transgenic mice and rabbits to assess the immunogenicity and protective efficacy of herpes epitopes against primary and recurrent infection.

Future herpes vaccines should use a needle-free mucosal application in which the epitopes are recognized by and stimulate the mucosal immune system. We recently found that synthetic peptide epitopes extended with an agonist of Toll-like receptor 2 (TLR-2), which is abundantly expressed on dendritic and epithelial cells of the vaginal and ocular mucosa, can lead to induction of protective immunity against herpes [123, 124]. Thus mucosal (topical ocular or intravaginal) immunization with self-adjuvanting lipid-tailed peptides bearing “asymptomatic epitopes” appears to have attractive practical and immunological features.

## Figures and Tables

**Table 1:** A sampling of past and ongoing preclinical and clinical vaccine trails.

Company Name	Product name	Phase of development*	HSV vaccine type	Mode of action^†^
Micro-Antigen Technologies, LLC	“Asymptomatic” Lipopeptide	PC	Peptide-based Self-adjuvanting	P&T
AlphaVax, Inc.	HSV Vaccine ALPHAVAX	PC	Alphavirus vector	P&T
Genocea Biosciences	HSV2 Vaccine GENOCEA	PC	Undisclosed	P&T
Henderson Morley plc	L-particles	PC	VLP	P
Henderson Morley plc	PREPS (previral DNA replication enveloped particles)	PC	VLP	P
JN International Medical Corporation	Genital Herpes Vaccine JN INTERNATIONAL	PC	Subunit	P
Juvaris Biotherapeutics, Inc	JVRS100 with Herpes Simplex Virus-2 Antigens	PC	Subunit gB, gDt, gH/gL JVRS-100 adjuvant	P
Mymetics Corporation	Herpes Simplex Virus Vaccine MYMETICS	PC	VLP	P
Sanofi-aventis	ACAM529	PC	Replication-defective virus	P&T
BioVex Inc.	ImmunoVEX HSV2 Vaccine	I	Live-attenuated virus	P
Pfizer Inc.	Genital Herpes DNA Vaccine PFIZER INC	I	DNA *via* PMED	T
AuRx, Inc.	Theraherb	III	Live-attenuated virus	T
GlaxoSmithKline plc	Simplirix	F	Subunit gD2 SBAS4 adjuvant	P
Acuvax Ltd (formerly Avantogen Limited)	HSV 2 ACUVAX	D	Live-attenuated virus GPI-0100 adjuvant	T
Antigenics Inc.	AG702	D	Subunit gB2 Human HSP-70 adjuvant	T
Antigenics Inc.	AG707	D	Subunit 32 peptides Human HSP-70 adjuvant	T
BioVex Inc.	ImmunoVEX HSV2/HPV Vaccine	D	HSV-2/HPV Combined	P&T
Celldex Therapeutics, Inc.	Dl5-29 Vaccine CELLDEX	D	Live, replication-impaired virus	T
Celtic Pharma Management L.P.	DISC Pro	D	DISC	P
Novartis AG	Genital Herpes DNA Vaccine NOVARTIS	D	DNA	T
Celtic Pharma Management L.P.	TAHSV	F	DISC	T
Eli Lilly&Co.	Resiquimod ELI LILLY	F	TLR agonist	T
GenVec Inc.	Herpes Simplex Virus Type 2 Vaccine GENVEC	NA	Adenovirus vector	P&T
Profectus bioSciences, Inc.	Herpes Simplex Virus Vaccine PROFECTUS BIOSCIENCES	NA	DNA with recombinant VSV boost	T
Vical Inc.	Herpes Simplex Virus Type 2 Vaccine VICAL	NA	DNA Vaxfectin adjuvant	T

The table recapitulates the majority of HSV vaccine candidates currently undergoing different phases of clinical trials, the companies that are conducting the trial, the phase of the trial, the type of vaccine, and the therapeutic approach. *PC: preclinical, I/III: phase I/phase III, D: discontinued, F: failed, NA: not available, ^†^P: prophylactic, and T: therapeutic.

**Table 2:** Herpes vaccine formulas used in clinical trials.

Type of HSV vaccine	Formulation	Strain	Route of administration	Clinical outcome
Live	Live HSV	Varies	Autoinoculation	(i) Unsuccessful (ii) Recurrence not affected (iii) Lesions at infection and injection sites [125, 126]
Live-attenuated	Recombinant R7020	HSV-1(F) and HSV-2(G)	Intramuscular	(i) Unsuccessful (ii) Poor immunogenicity (iii) Adverse effects in HSV-1 seropositive individuals [127, 128]
Whole inactivated	Heat inactivated (Lupidon G and H)	HSV-2(Silow) and HSV-1(L3)	Subcutaneous	(i) Statistically significant effect on recurrence of genital and facial herpes [129–131]
	Formalin inactivated	—	—	(ii) No significant difference in recurrence compared to placebo [132]
Inactivated subunit	Skinner: Ac NFU_1_, (S-) MRC	HSV-1 (Troisbell)	Subcutaneous	(i) Some statistically significant results in vaccinated males (ii) No consistent efficacy or immunogenicity [133]
Recombinant subunit (glycoproteins)	Chiron gD2gB2-MF59	HSV-2	Intramuscular	(i) No significant effects on recurrence or shedding of virus [17]
	GlaxoSmithKline gD2-Alum MPL	HSV-2	Intramuscular	(ii) Fewer recurrences (iii) Higher antibody and gD2-specific EIA titers compared to placebo [1, 134]
Disabled infectious single cycle (DISC)	TA-HSV-2	HSV-2(25766) HSV-1(HFEM) HSV-1(SC16) HSV-1(KOS) HSV-1(*ts*Q26)	?	(i) Good immunogenicity in early clinical trials (ii) Unsuccessful phase II trials (iii) No significant differences in recurrences or asymptomatic shedding compared to placebo [135]

The table summaries past and present HSV vaccine formulations, HSV-1/2 strains used, route of administration, and clinical outcomes.
